# Baby Clinic Experiences

**Published:** 1920-07-03

**Authors:** 


### THE EDITOR'S LETTER-BOX.

### Baby Clinic Experiences.

*To the Editor of* The Hospital.

Sir,—One can sympathise with all the difficulties which Dr. C. J. Coleman, of Lincoln, described in your issue of last week, but more particularly with that of persuading the mother that under average conditions it is not a wise and often beneficial procedure to wean her child at one to two months. It is a surprising but certain fact that one repeatedly hears the excuse that a doctor has told the mother that her milk is apparently unsuitable for her child, and although such a statement may not always represent the whole truth concerning the medical advice she has received, I heartily agree that frequently the local medical profession have been to blame for not more insistently encouraging breast-feeding. There is, however, another cause—namely, the form of advertisement published by not a few manufacturers of artificial infant foods. Descriptions of these products and of the exact ways in which they are reputed to meet every infantile dietetic requirement are given the fullest prominence, and in practice greatly obscure from the mother the fact that they are in reality but a second line of defence. One has often caught my attention. "Mothers, nurse your babies! If you can't, use ——." At first sight it is sound advice, but it is also a subtle form of temptation.

I am, Sir, yours faithfully,

M.B., D.P.H.

